# Identification and Validation of a Putative Polycomb Responsive Element in the Human Genome

**DOI:** 10.1371/journal.pone.0067217

**Published:** 2013-06-21

**Authors:** Hemant Bengani, Shweta Mendiratta, Jayant Maini, Dasari Vasanthi, Hina Sultana, Mohsen Ghasemi, Jasmine Ahluwalia, Sowmya Ramachandran, Rakesh K. Mishra, Vani Brahmachari

**Affiliations:** 1 Dr. B. R. Ambedkar Centre for Biomedical Research, University of Delhi, Delhi, India; 2 Centre for Cellular and Molecular Biology (CSIR), Hyderabad, Andhra Pradesh, India; CNRS, France

## Abstract

Epigenetic cellular memory mechanisms that involve polycomb and trithorax group of proteins are well conserved across metazoans. The *cis*-acting elements interacting with these proteins, however, are poorly understood in mammals. In a directed search we identified a potential polycomb responsive element with 25 repeats of YY1 binding motifthatwe designate PRE-PIK3C2B as it occurs in the first intron of human *PIK3C2B* gene. It down regulates reporter gene expression in HEK cells and the repression is dependent on polycomb group of proteins (PcG). We demonstrate that PRE-PIK3C2B interacts directly with YY1 *in vitro* and recruits PRC2 complex *in vivo*. The localization of PcG proteins including YY1 to PRE-PIK3C2B in HEK cells is decreased on knock-down of either *YY1* or *SUZ12*. Endogenous PRE-PIK3C2B shows bivalent marking having H3K27me3 and H3K4me3 for repressed and active state respectively. In transgenic *Drosophila*, PRE-PIK3C2B down regulates mini-white expression, exhibits variegation and pairing sensitive silencing (PSS), which has not been previously demonstrated for mammalian PRE. Taken together, our results strongly suggest that PRE-PIK3C2B functions as a site of interaction for polycomb proteins.

## Introduction

During development, the transcription status of the genes is maintained from embryonic to adult stages through mitotic or cellular memory mechanisms, which leads to the establishment of different cell lineages. The polycomb (PcG) and trithorax (TrxG) group of proteins, discovered in the context of regulation of homeotic genes in *Drosophila*, function through their interaction with chromatin as multi-protein complexes [1]. These genes are well conserved in metazoans and their mammalian counterparts are transcription regulators that help in maintaining cell identity through chromatin modification [2], [3]. The members of the complex catalyze histone modification; like EZH2 in PRC2, confer H3K27me3 mark which in turn leads to the recruitment of PRC1 complex and repression of the target gene expression [4], [5]. Historically, PcG members are known to maintain a repressed state of gene expression while TrxG members maintain an active state. Recently, it is demonstrated that EZH1, a paralogue of EZH2 is associated with H3K4me3 and RNA Pol II in undifferentiated mouse myoblasts [6]. Thus, the functional diversity of polycomb complexes to meet the demand of contextual function in mammalian cells is becoming increasingly evident [7], [8]. Similar functional diversity is observed in the cis-elements interacting with polycomb complexes. In *Drosophila*, PcG/TrxG proteins regulate their target genes by binding to specific DNA elements called Polycomb/trithorax response elements (PRE/TREs) which interact with both activating and repressive complexes and PRE/TREs recruit PcG/TrxG complexes at multiple loci as seen on polytene chromosomes [9], [10]. Analysis of the known PREs has revealed the presence of binding sites, often in multiple copies, for several DNA-binding proteins, such as Pleiohomeotic (PHO) and Pleiohomeotic-like (PHOL) [11], GAGA factor (GAF)/Pipsqueak (PSQ), Zeste and DSP1 [12], [13]. Recent work has suggested the possible additional roles for proteins such as the corepressor CtBP, the DNA binding factor Grainyhead(GRH) and members of the Sp1/KLF family, which are also DNA binding factors [14]–[16].

Pleiohomeotic (PHO), a PcG protein from *Drosophila*, functions in this recruitment as it is known to bind DNA specifically and is involved in the recruitment of PcG complex [17], [18]. PHO binding sites are found in many PRE sequences and mutation of either PHO protein or its binding site reduces PcG silencing, indicating that it is an important component of PcG repression mechanism [17], [19], [20]. YY1 is the functional homologue of PHO identified in mammals, which is known to repress transcription in a PcG dependent manner and compliments the loss of PHO in mutant flies in multiple functions [21]. siRNA mediated down regulation of YY1 results in the loss of recruitment of EZH2 and concomitantly the absence of H3K27me3 at the site [22]. The conservation in binding motif together with the existing biochemical evidence, suggests that YY1 plays an important role in the recruitment of vertebrate PcG and TrxG complexes [22]–[24]. Although, a number of recent studies have shown that apart from YY1, there might be other recruiters for the polycomb complex. Recruitment of PcG complexes via non-coding RNAs and the role of GATA-1 in recruiting PRC2 complex during erythroid maturation are also reported [25], [26]. Transcription factors such as Runx1 and CBFβ recruit PRC1 complexes independent of the PRC2 complexes in the lymphocytes [27].

Though mammalian homologues of several PcG and TrxG proteins are known, PRE/TRE-like function is attributed only to a few *cis*-regulatory elements [22], [23], [28]. Recently PRE-kr that regulates the expression of mouse MafB/Kreisler gene and D11.12 in the region between HoxD11 and HOXD12 in human embryonic cells were reported [29], [30]. The misexpression of *MafB* gene due to inversion of a large sequence led to the identification of PRE-kr in mice, while the selective enrichment of H3K27me3 and PcG proteins and its correlation with MNAse sensitivity led to the identification of *HOXD11-12* PRE in human embryonic cells [29], [30]. PRE-kr was tested for its function in ectopic context in *Drosophila* miniwhite reporter as well as in transgenic mice [29]. Altered epigenetic marking has been identified as the molecular basis of FSHD (facio scalpulohumeralmuscular dystrophy). A decrease in the number of repeats of PRE-like sequences in the upstream region within the D4Z4 repeats reduces its association with PRC complex leading to a decrease in repressive epigenetic modification [31]. In a candidate approach based on data on localization of H3K27me3 and the PRC members, putative PREs were identified in human T cells and PRC2 mediated repression of transcription of the genes in the region was also demonstrated [32]. The correlation between temporal epigenetic modification with the successive expression of Hox genes during mouse development was observed by Soshnikova and Duboule [33], indicating that transcriptional competence of Hox genes during development is brought about by epigenetic modifications.

Our goal was to search for potential PRE/TRE like sequences in the human genome, therefore we carried out *in silico* analysis to retrieve genic regions with high density of YY1 consensus motif in the human genome. We identified a 1 kb sequence with 25 mer repeating unit containing the YY1 binding site repeated 25 times at the firstintron of PIK3C2B gene and studied its regulatory function on reporter gene expression. The repression of the reporter as well as the endogenous *PIK3C2B* is dependent on PRC proteins. Further we validate this sequence for its ability to function as PRE/TRE in transgenic *Drosophila* and detect genetic interaction with PcG and TrxG mutants. Based on these results we discuss the implied PRE/TRE like function of PRE-PIK3C2B.

## Materials and Methods

### Plasmids, Antibodies and Primers/Oligonucleotides

pcDNA3.1 GFP was obtained from Sanjeev Khosla, CDFD, Hyderabad, India. pHIS-YY1 [34], pRetroSuper-SUZ12 [35], pSUPER-YY1 [36] was a kind gift from Thomas Shenk, USA, Kristian Helin, Denmark and Mark Featherstone, Singapore, respectively.

Antibodies against YY1 (ab43058), SUZ12 (ab12073), EED (ab4469), EZH2 (ab3748), H3K4me3(ab8580) and H3K27me3 (ab6002) were purchased from Abcam. Antibody against EZH2 was purchased from Active Motif (Cat. No. 39875). Anti-Brahma antibodies was a kind gift from Madan Mohan Chaturvedi, Delhi University. Antibodies against Polycomb, trithorax and Polyhomeotic [37] was gifted by Giacomo Cavalli, CNRS, France and anti-PHO [19] was gift from Judith Kassis, USA.

Primers **P1** (*5*′*TTGACAAATGCAATAACAAGGC-3*′), **P2** (*5* ′*-TCCCTTGTGTCCCGTTGTAATC-3*′), **P3** (*5* ′*-GGTATTCTCACATGTCAAAA-3*′ *) and* **P4** (*5* ′*-GGATCCTAGTGCAAGCTG-3*′), were used for PCRs as required. P1-P4 primers were used for amplification of PRE-PIK3C2B from human genomic DNA. **Oligo25-F** (*5* ′*-AGTGAAGCCATCAT GTGAGAATACC-3*′) and **Oligo25-R** (*5* ′*-GGTATTCTCACATGATGGCTTCACT-3*′); **OligoΔYY1-F** (*5* ′*-AGTGAAGTGAGAATACC-3*′) and **OligoΔYY1-R** (*5* ′*-GGTATTC TCACTTCACT-3*′) were used in binding studies by EMSA. **WNT1-F** (*5* ′*-CCAGCGCCGCAACTATAAGAG-3*′) and **WNT1-R** (*5* ′*-CGCAGTCTGGCTTTAA CAACCC-3*′); **RNAP-F **(*5* ′*-GGTTTACCCACGACTCTGGCTC-3*′)and **RNAP-R** (*5* ′*-GCTTCTGAGCAGCGAACTCG-3*′); **GDAP1-F** (*5* ′*-GCTTTCCAGTCGCAGACC-3*′) and **GDAP1-R** (*5* ′*-GCCTCTCAGCCATCTTGG-3*′); **GAPDH-F** (*5* ′*-TACTAGCGGTTT TACGGGCG-3*′) and **GAPDH-R** (*5* ′*-TCGAACAGGAGGAGCAGAGAGCGA-3*′)were used in chromatin-immunoprecipitation(ChIP) assays.

### In silico analysis

Genic sequences along with 15 kb each of 5′and 3′ flanking regions of genes with altered expression in ALL patients [38] were downloaded from NCBI build 34. A PERL script was written to identify YY1 binding sites in the sequences.

### Plasmid construction, transient transfection and expression analysis

1 kb of the PRE-PIK3C2B region was PCR amplified with P1-P4 primers from human genomic DNA and cloned into pcDNA-GFP vector in the Nru1 and Sma1 site to generate pPRE-PIK3C2B/_UP_GFP and pPRE-PIK3C2B/_DN_GFP respectively. To generate plasmid pc(25mer)11_UP/DN_, PRE-PIK3C2B was used as the template and the product was amplified using Oligo25-F and Oligo25-R as primers mapping within the repeat region and the pool of amplicons were cloned in pTZR57R/T and sequenced. Insert from the clone with the tandem ligation of the Oligo25 mer (11 times) was cloned in the Nru1 and Sma1 site of pcDNA-GFP vector to generate pc(25 mer)11_UP_ and pc(25 mer)11_DN_ respectively. To generate plasmid pc(ΔYY1)11_UP/DN_, OligoΔYY1-F and OligoΔYY1-R were annealed by heating at 95°C for 5 minutes and kept at room temperature to cool slowly. Annealed OligoΔYY1 was self-ligated and the pool of self ligated OligoΔYY1 was used as template for PCR using OligoΔYY1-F and OligoΔYY1-R as primers. The amplicons were cloned in pTZR57R/T and sequenced. Insert from the clone with the tandem ligation of the OligoΔYY1 (11 times) was cloned in the Nru1 and Sma1 site of pcDNA-GFP vector to generate pc(ΔYY1)11_UP_ and pc(ΔYY1)11_DN_ respectively. Transfection of HEK293 cells with the vectors generated and also the siRNA constructs were carried out using Transpass D2 (New England Biolabs) as per the manufacturer's protocol. The cells were analysed for GFP reporter expression after 48 hours by using Flow cytometry (Guava Technology, USA).

Expression of endogenous PIK3C2B in HEK293 cells was analyzed by qPCR in absence and presence of siRNA YY1 and siRNA SUZ12 using the primers PIKb1F (5′-CCCCACTCTTCAACTTCAGG-3′) and PIKb1R (5′TCTGG CTGGTGGAAATTAGG-3′). Transfection of pSUPER-YY1 and pRetroSuper-SUZ12 was carried out using Lipofectamine2000 (Invitrogen,USA) as per the manufacturer's protocol, cDNA was synthesized from isolated RNA (Trizol, Invitrogen) from cells after 48 hours after transfection, *GAPDH* was used as internal control.

### EMSA (Electrophoretic Mobility Shift Assay)

Gel mobility-shift assay and preparation of nuclear extract from HEK293 cells were carried as described by Mishra et al., [20] with slight modifications. Nuclear extract with 5 µg of total protein or 200 ng of purified YY1 was incubated with 1 µg of poly (dI-dC) and 1 µg of tRNA in 20 µl in 25 mM HEPES pH 7.6, 1 mM KCl, 0.1 mM EDTA, 1 mM DTT, 0.1 mM PMSF, 10% glycerol and 100 mM NaCl for 10 minutes at room temperature before adding γ^32^PATP-labelled double-stranded Oligo25 mer (2 ng). The γ^32^PATP end-labeling reaction was performed using Polynucleotide Kinase at 37°C for 60 minutes. After adding the labeled probe, the mixture was incubated at room temperature for 20 minutes. Electrophoresis was carried out in 5% acrylamide-bisacrylamide (40∶1) gel containing 4% glycerol in 0.5× TBE. For competition experiments, the unlabeled DNA probes were added at the same time as the labeled probes. To detect the binding of YY1, labeled double-stranded Oligo25 mer and purified YY1 protein were incubated with anti-YY1 antibody and binding was detected using Phosphor imager (Typhoon 9210, GE Healthcare).

### Chromatin immunoprecipitation assay (ChIP) and qPCR

Cross-linked chromatin was prepared from transgenic *Drosophila* embryos and ChIP was performed as described previously [1]. SYBR green chemistry based qPCR analysis was performed to assess the enrichment of PRE-PIK3C2B computed as percentage of input DNA. Non-immune rabbit IgG was used as the control. Similarly ChIP followed by qPCR in HEK293 cells was carried out following transfection of HEK293 cells with pRetroSuper-SUZ12 and pSUPER-YY1 as described earlier [39]. The results were analysed for statistical significance using Student's t-test.

### Generation of transgenic flies and genetic interaction studies

The PRE-PIK3C2B fragment was PCR amplified from human genomic DNA with primers P1 and P4 cloned into pGEM-T Easy Vector System (Promega) and sequenced. The fragment re-cloned in pLML vector at the EcoR1 and HindIII restriction site is flanked by loxP sites. The insert with the loxP sites from pLML vector was cloned at XhoI site upstream of miniwhite promoter in pCasPeR vector with miniwhite reporter gene. The construct was then injected into embryos from w^1118^ Canton-S strain [40]. Once the balanced stocks of all the lines were made, a flipped out version for each line was generated by crossing homozygous males of transgenic lines with virgins expressing Cre recombinase (Bloomington stock-766). Stocks were balanced and the absence of PRE-PIK3C2B was confirmed by PCR. Details of crosses used in genetic interaction studies in different mutant backgrounds are available on request. For eye color comparison, sex and age matched flies were imaged in the same frame and image processing was under the same conditions.

To analyze the effect of Pleohomeotic on miniwhite expression, quantitative PCR for miniwhite expression was carried out. Age matched adult males of the following genotypes were compared: (i) w^1118^; PI-17/+; +/+, (ii) w^1118^; PI-17/+; Phob/+ (iii) w1^118^; ΔPI-17/+;+/+(iv) w^1118^; ΔPI-17/+; Phob/+. Δ indicates the flip out of PRE-PIK3C2B using Cre recombinase. RNA was extracted from the adult flies, cDNA preparation and qPCR protocols were as described for other genes in the earlier section. Exon spanning primers (forward primer 5′- ATGGCGGCAGCTGGTCA-3′ and reverse primer 5′– GCGGCGATCGAAAGGCAA- 3′) were used for qPCR.

## Results

### Identification of putative regulatory sequences

To detect putative PRE/TRE in the human genome in silico, we selected genes that show altered expression in acute lymphoblastic leukemia (ALL) with t(4∶11) translocation, because the translocation partner MLL on chromosome 11 is a homeotic regulator that shares homology with *Drosophila* trithorax gene [41]. Thus the genes with altered expression in the background of MLL translocation could be the direct or indirect targets of MLL. The expression data of Armstrong et al., [38] was used to retrieve 100 genes each from over and under expressed transcripts from a microarray analysis involving MLL translocation. The genic sequences along with 15 kb each from upstream and downstream of the gene was retrieved for analysis.

We considered the density of YY1 binding motif per 10.00 kb along the length of the selected sequences as a criterion for identification of putative PRE. The top ten genes with high density of YY1 motifs were identified where PIK3C2B was at the top of this list (Table S1). Since *PIK3C2B* is an important signaling gene, we selected it for experimental validation. We refer to this sequence as PRE-PIK3C2B, which occurs in the first intron of the gene and is a 1 kb sequence containing 25 repeats of YY1 binding consensus sequence (NC000001: 204439945–204440999; Fig. S1).

### PRE-PIK3C2B is a regulatory element

**Figure 1:**
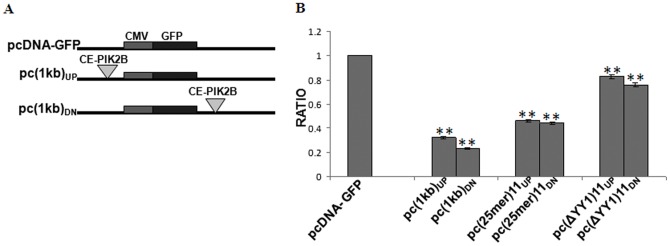
Effect of PRE-PIK3C2B on transcription. A. Part of the vectors used in transfection assays: CMV represents the promoter, GFP is the reporter present in all the constructs. pcDNA3.1-GFP is control plasmid without PRE-PIK3C2B, pc(1 kb)UP is pPRE-PIK3C2B/UP-GFP and pc(1 kb)DN is pPRE-PIK3C2B/DN-GFP with PRE-PIK3C2B (inverted triangle) cloned upstream and downstream of the reporter respectively. B. GFP expression indicated as ratio relative to that from pcDNA3.1GFP. pc(25 mer)11UP and pc(25 mer)11DN are 25 mer oligo corresponding to repeating unit of PRE-PIK3C2B cloned upstream and downstream of the GFP reporter respectively. pcΔYY1UP and pcΔYY1DN are repeating unit without YY1 motif cloned upstream and downstream of the GFP reporter respectively. Error bars, S.E.M of assay triplicate is shown. (**) p-value<0.005, n = 3

PRE-PIK3C2B was tested for its effect on CMV promoter activity in a GFP reporter construct derived from pcDNA3.1GFP. We generated two constructs; one in which PRE-PIK3C2B was cloned upstream of the promoter and the other where it is downstream to the poly(A) signal of the reporter; pPRE-PIK3C2B/_UP_-GFP and pPRE-PIK3C2B/_DN_-GFP respectively and transfected them into HEK cells (Fig. 1A). After normalizing, the GFP expression was reduced by 70% in pPRE-PIK3C2B/_UP_-GFP and 80% in pPRE-PIK3C2B/_DN_-GFP, indicating that PRE-PIK3C2B negatively regulates the reporter expression when it is either upstream to the promoter or downstream to poly(A) signal (Fig. 1B).

To narrow down the regulatory activity within PRE-PIK3C2B, we generated pc(25 mer)_11_clones with 11 repeats of 25 mer containing one GCCATCAT motif for YY1 binding in each repeating unit and transfected them into HEK cells. We observed repression of GFP expression by 54% and 56% when cloned upstream and downstream of reporter (Fig. 1B). Further, on transfection of HEK cells with pcΔYY1 in which YY1 motif is deleted, there was no significant effect on transcription compared to the control. Therefore, YY1 motif is essential for transcription repression (Fig. 1B).

### PRE-PIK3C2B directly interacts with YY1

**Figure 2:**
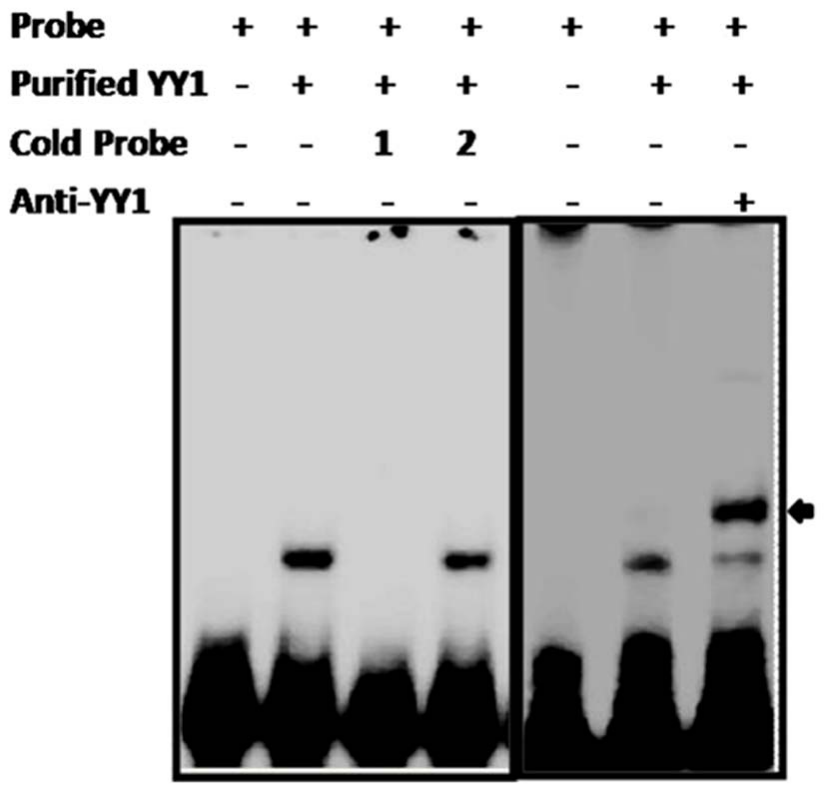
Directinteraction of YY1 with PRE-PIK3C2B. Labeled 25 merOligo corresponding to repeating unit of PRE-PIK3C2B (5′AGTGAAGCCATCATGTGAGAATACC3′) was used as the probe in gel mobility shift assay with purified His-YY1. Cold probe 1 is competing unlabeled cognate (Oligo25 mer), 2- (OligoΔYY1) repeating unit without YY1 recognition sequence (5′AGTGAAGTGAGAATACC3′). Each competing oligo was used at a concentration 100× higher than the labeled probe. The super shift with anti-YY1 is shown in the last lane (arrow).

We detected the direct interaction of purified recombinant YY1 protein with end labeled Oligo25 mer. The specificity of binding is shown by the lack of competition by oligonucleotide with deletion of YY1 binding site (OligoΔYY1), while unlabeled Oligo25 mer could compete out the binding (Fig. 2). Further, retardation of the probe on incubation with antiYY1 antibody is also seen (Fig. 2). We detected the interaction of the Oligo25 mer (repeat from PRE-PIK3C2B) with proteins from nuclear extract of HEK cells (Fig. S2) and the competition with unlabeled oligos with and without YY1 motif indicated the specificity of the interaction(Fig. S2). Repeated attempts at super-shift assays with anti-YY1 antibody did not yield results. To further confirm the YY1 mediated regulation, we studied the effect of depletion of YY1 on reporter expression in vivo.

### Interaction of PRE-PIK3C2B with PcG proteins

**Figure 3:**
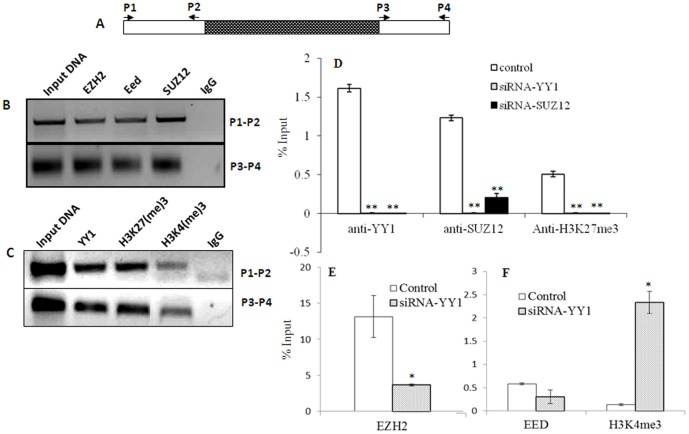
Interaction of PcG proteins with PRE-PIK3C2B in HEK cells. The experiments were carried out in HEK293 ells. A-Diagrammatic representation of the endogenous PRE-PIK3C2B region with primers (arrows) used for PCR and the 25 mer repeating unit (filled box). B and C- ChIP with antibodies indicated. Input is 20% of sonicated chromatin. D-Quantitative PCR for PRE-PIK3C2B following ChIP in control cells and cells transfected with siRNAYY1 and siRNA SUZ12. E-Interaction of EZH2 with PRE-PIK3C2B estimated by qPCR in absence and presence of YY1siRNA: F-Localization of EED and the H3K4me3 at PRE-PIK3C2B in absence and presence of siRNAYY1. Error bars: S.E.M of assay in triplicate, * p<0.05, **p-value<0.005,n = 3. The qPCR profile for positive and negative controls are shown in Figs. S3 and S4.

The PRC complexes localize at PRE, and the methyl transferase activity of EZH2 in PRC2 complex brings about H3K27me3 modification. Therefore we analysed the methylation status and also the localization of PRC2 members at PRE-PIK3C2B in HEK293T cells (Fig. 3). Following Chromatin immunoprecipitation with the specific antibodies, PRE-PIK3C2B was detected using the primers P1-P2 and P3-P4 mapping within PRE-PIK3C2B (Fig. 3B and C). The primers spanning the repeat motif could not be used as it resulted in multiple amplicons. We detect the interaction of YY1, SUZ12, EZH2 and EED with endogenous PRE-PIK3C2B (Fig. 3B,C). In addition, PRE-PIK3C2B is also pulled-down by anti-H3K27me3 as well as anti-H3K4me3 (Fig. 3C). Since we observed similar results with both the primer sets, we used P3–P4 in subsequent qPCR experiments.

On knock-down of YY1 and SUZ12 using siRNA constructs, H3K27me3 is lost and localization YY1and SUZ12 is significantly reduced at PRE-PIK3C2B (Fig. 3D). We confirmed the localization of EZH2 and EED also at the endogenous PRE-PIK3C2B which was decreased on knock-down of YY1 (Fig. 3E, F). The knock down of YY1 results in the loss of H3K27me3 and increase in H3K4me3 mark (Fig. 3D, F).

We used GAPDH and GADP1 respectively as negative and positive controls for YY1 (Fig. S3) [42]. PRE-PIK3C2B shows significant interaction with YY1, SUZ12, EZH2 and EED. We used WNT and RNAP respectively as positive and negative controls for SUZ12, EZH2 and EED, based on previous reports (Fig. S4) [43], [44].

### Effect of Polycomb proteins on transcription

**Figure 4:**
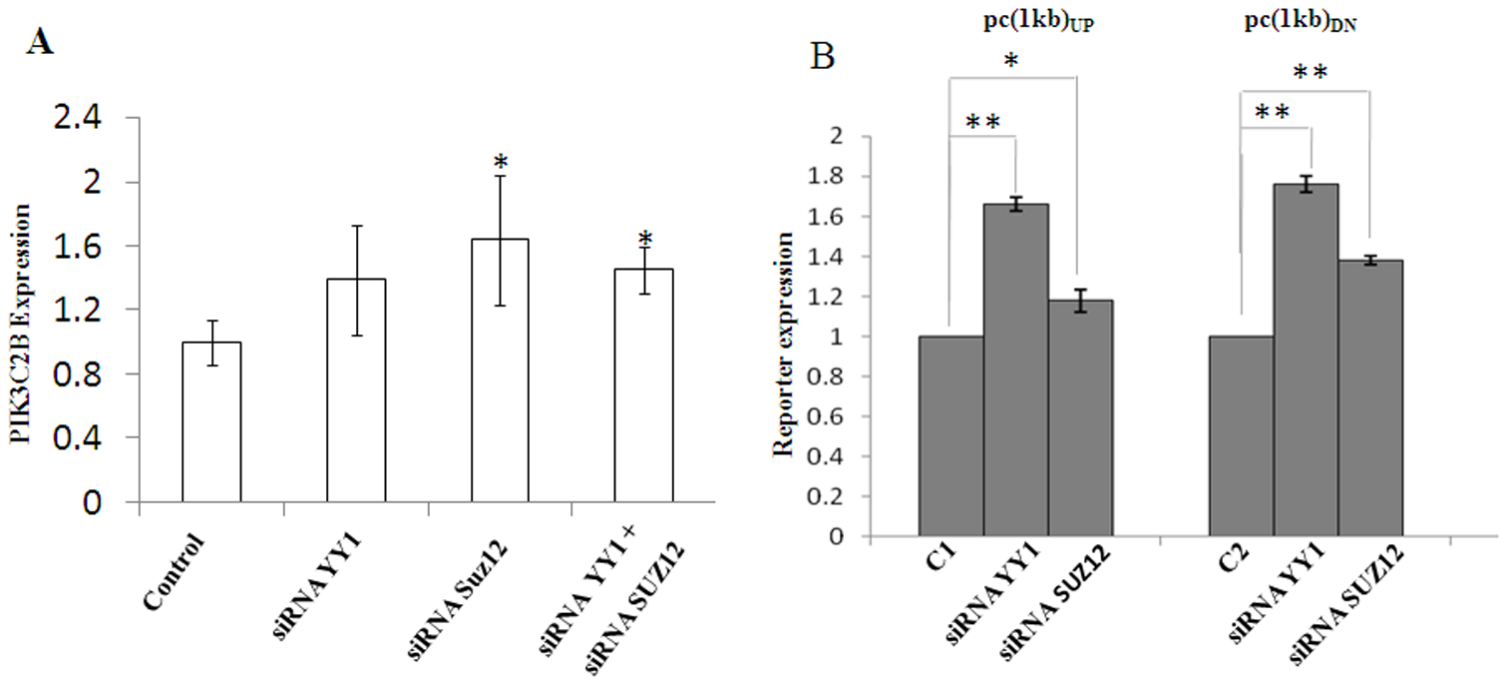
YY1 and SUZ12 dependent transcriptional repression by PRE-PIK3C2B. A-Expression of endogenous *PIK3C2B* in HEK cells analysed by qPCR. The expression is increased in presence of siRNAYY1 and siRNASUZ12. Expression in untransfected HEK cells is taken as control. B- Effect on reporter gene expression. The nomenclature for vectors is same as in Figure 1. Ratio of GFP expression in pcDNA-GFP, pc(1 kb)UP or pc(1 kb)DN with and without co-transfection with siRNA YY1 and siRNA SUZ12 is shown on the Y-axis. Error bars, S.E.M of assay in triplicate is shown * p-value<0.05, ** p-value<0.005, n = 3.

We analyzed the effect of PRC2 binding to PRE-PIK3C2B on the expression of endogenous *PIK3C2B* in HEK293T cells and also the reporter gene in separate experiments (Fig. 4). We compared the expression of endogenous PIK3C2B in presence and absence of YY1 and SUZ12 (Fig. 4A). The knock down of YY1 is nearly 90% after 48 hours (Fig. S5). We observe a significant reversal of repression on knock-down of both the proteins together and not knockdown of YY1 alone. This may relate to the affinity of the complex as discussed later. Similarly the GFP reporter constructs with PRE-PIK3C2B (pPRE-PIK3C2B/_UP_-GFP and pPRE-PIK3C2B/_DN_-GFP) were co-transfected with siRNA constructs and a reversal of repression was observed (Fig. 4B). Thus, our results show that the interaction of *PRE-PIK3C2B* with PRC2 complex leads to transcription repression in the cells. This implies that *PIK3C2B* could be one of the *in vivo* targets of PRE-PIK3C2B. The increase in transcription of *PIK3C2B* is significant but modest. It is important to note that reversal of repression does not mean activation and additional transcription factors may be required for activation of *PIK3C2B*. The localization of PRC2 and the bivalent marking at PRE-PIK3C2B, strongly suggest that PRE-PIK3C2B we describe here could indeed be a potential PRE/TRE in the human genome. We tested if PRE-PIK3C2B can function as a PRE in *Drosophila*.

### PRE-PIK3C2B functions as PRE/TRE in *Drosophila melanogaster*

**Figure 5:**
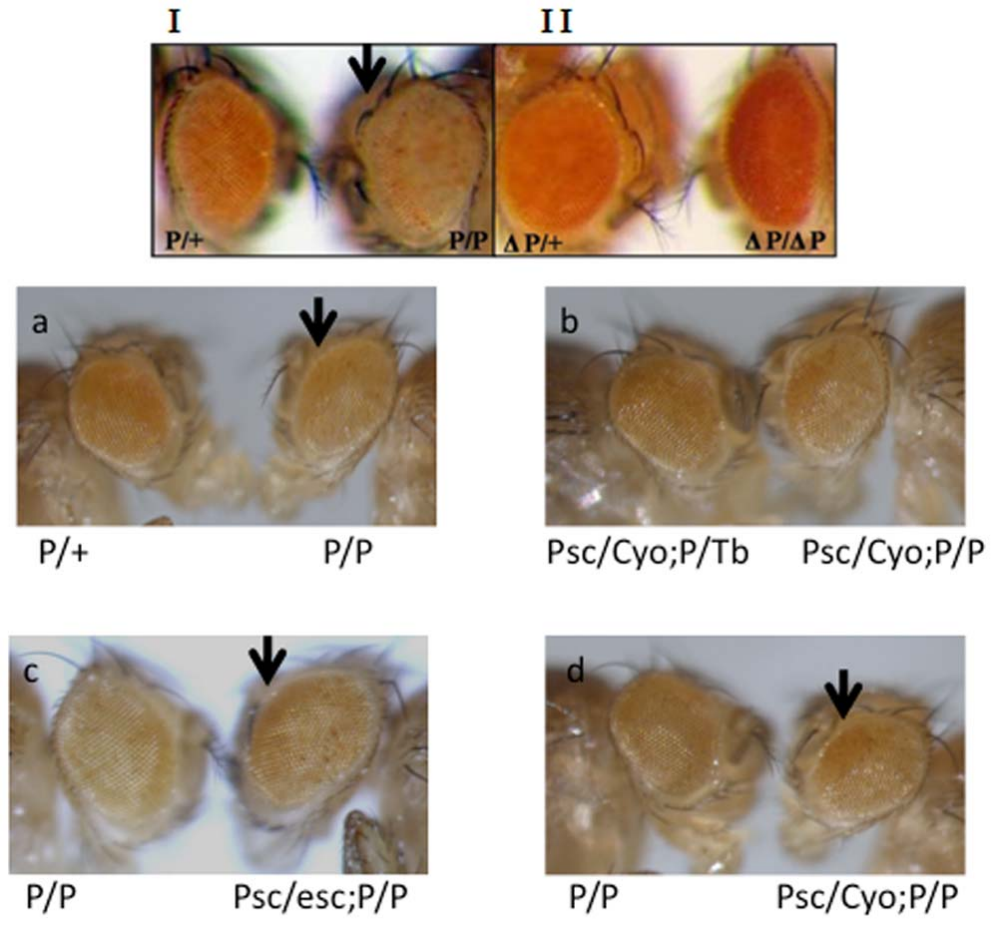
Pairing sensitive silencing (PSS) of miniwhite expression. Eye pigmentation due to miniwhite expression in transgenic flies of PI-17 line are shown. I- P/+ and P/P, heterozygous and homozygous transgenic lines, homozygous flies show PSS; II- ΔP/+ and ΔP/ΔP, are transgenic flies where PRE-PIK3C2B is flipped-out by crossing with Cre flies. The frames a-d show rescue of PSS in different genetic backgrounds as indicated. Arrow in frame a marks PSS in homozygous flies, arrow in frame c and d mark the rescue of PSS.

We generated 16 independent transgenic fly lines with PRE-PIK3C2B flanked by loxP sites, cloned upstream of the miniwhite promoter in the P-element vector pCaSpeR (Fig. S6 and Table S2). Miniwhite gene was down-regulated in the transgenic flies and the repression was reversed when PRE-PIK3C2B was flipped out by Cre recombinase (Fig. 5). There are several attributes of PRE/TRE sequences that are assayed in *Drosophila*. Among them, PRE/TRE placed near miniwhite gene is expected to show variegation and pairing sensitive silencing [45]–[47]. We observed variegation in almost 50% of the transgenic fly lines (Fig. 5I and Table S2). The variegation indirectly indicates that the activity state of miniwhite is transmitted through cell division, an attribute of a classical PRE/TRE in *Drosophila* [12]. Thus, our results suggest that PRE-PIK3C2B fulfills the maintenance function expected of a PRE/TRE. In addition, transgenic line PI-17 where the PRE-PIK3C2B integration was mapped on the third chromosome, exhibits pairing sensitive silencing (PSS) or pairing dependent silencing(PDS) when the PRE-PIK3C2B is in homozygous state (Fig. 5I). To the best of our knowledge this is the first report where PSS is observed for a mammalian PRE in *Drosophila*. The role of flanking endogenous sequence in PSS is ruled out as the expression of miniwhite is normal on flip out of the PRE-PIK3C2B sequence (Fig. 5II). Further we tested the effect of trans-heterozygotes on PSS (Fig. 5a-d). In the background of Psc (posterior sex comb) and esc(extra sex comb) in double heterozygous state, PSS is reduced and eye pigmentation is increased (Fig. 5c).

### Genetic interaction of PRE-PIK3C2B with PcG/TrxG members

**Figure 6:**
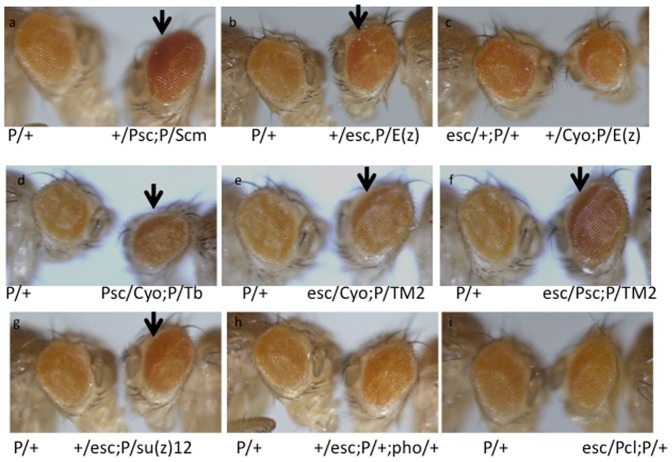
Genetic interactions of PRE-PIK3C2B with PRC genes in transgenic flies. Effect of PRE-PIK3C2B in the background of different PcG mutations on rescue of eye pigmentation is shown: cases of rescue of eye colour are marked with an arrow; trans-heterozygotes show better recovery of eye pigmentation. We have used same conditions of image processing in all the cases.

**Figure 7:**
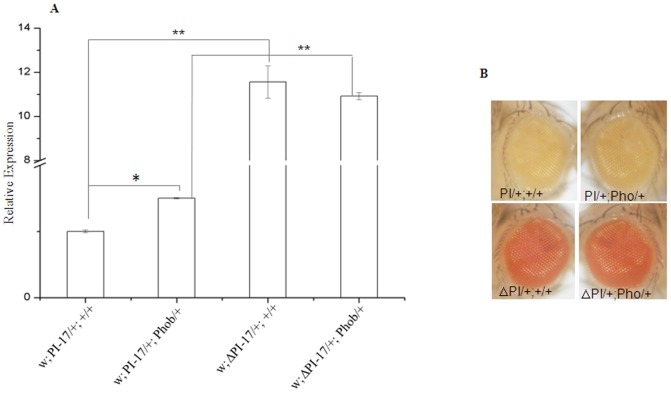
Effect of Pho mutation on PRE-PIK3C2B mediated repression. A- Expression of miniwhite mRNA by qPCR. The genetic background is indicated below the histograms.* p<0.05, **p<0.005. B- comparison of eye pigmentation between transgenic flies with and without PHO mutation. ΔPI indicates the lines where PRE-PIK3C2B is flipped out.

**Figure 8:**
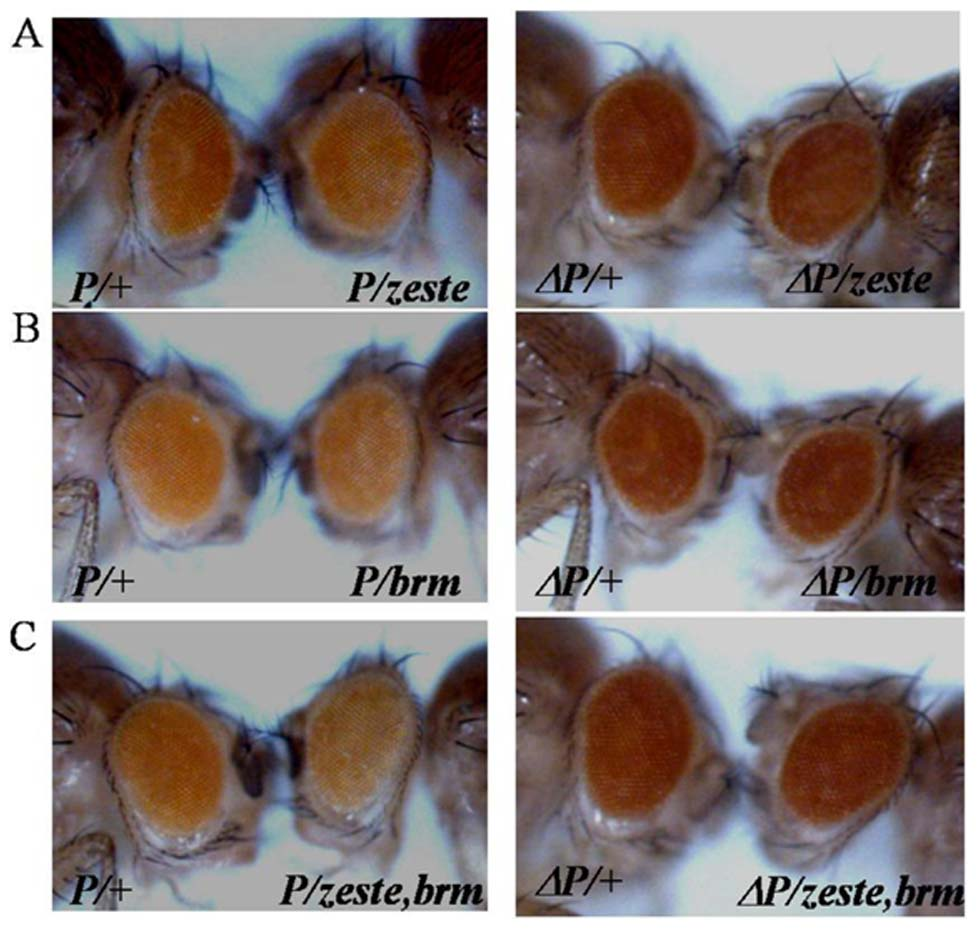
Genetic interactions of PRE-PIK3C2B with Trx genes in transgenic flies. Effect of PRE-PIK3C2B in the background of different TrxG mutations on eye colour is shown: single mutants zeste(A), brm (B) and double mutant zeste and brm(C). The left panel shows the transgenic flies heterozygous for PRE-PIK3C2B and the right panel are transgenic flies deleted for PRE-PIK3C2B in similar background. We have used same conditions of image processing in all the cases.

We analysed the genetic interaction between PRE-PIK3C2B transgenics and different PcG or TrxG mutations (Table S3 and S4).We tested the effect of mutations in several PcG proteins on PRE-PIK3C2B (Fig. 6). The rescue of eye colour was observed in the background of trans-heterozygotes of Psc and Scm, E(z) and esc, esc/Psc, esc and Su(z)12. Since PRE-PIK3C2B is enriched in Pho binding sites, we tested the effect of Pho mutation in PI-17 line by estimating the expression of miniwhite gene by qPCR (Fig. 7). We compared miniwhite expression in adult flies with deletion of PRE-PIK3C2B in the presence and absence of Pho mutation with that in PRE-PIK3C2B transgenics in similar genetic background. A significant rescue of miniwhite expression in flies with PRE-PIK3C2B was observed in Pho mutants, while in the PRE-PIK3C2B deleted lines there was no difference between miniwhite expression with or without Pho mutation (Fig. 7). The increase in miniwhite transcript level is clearly shown by qPCR(Fig. 7A). We analysed the interaction of TrxG genes with PRE-PIK3C2B and found that the reduction in the zygotic dosage of brm and zeste enhance PRE-PIK3C2B mediated repression of miniwhite gene resulting in further decrease in the eye color (Fig.8A and B), which was higher in zeste/+; brm/+ double heterozygote background (Fig. 8C). We detected interaction of PRE-PIK3C2B with PcG proteins in the transgenic flies by ChIP assays (Fig. S7). Pleiohomeotic (pho), Polycomb and Brahma were associated with PRE-PIK3C2B in the transgenic embryos.

## Discussion

We aimed at identifying putative PREs in the human genome based on *in silico* analysis for the occurrence of common motifs for binding of polycomb complexes. The gene set we selected for analysis shows over or under expression in ALL patients with t(11∶4) translocation and detected a high density of YY1 binding motif in the first intron of *PIK3C2B*. The other recently identified, PRE-kr, that regulates expression of mouse MafB/Kreisler gene, D11.12, the region between *HoxD11* and *HOXD12* identified in human embryonic cells and PRE in FSHD also contain YY1 binding motif [29], [30], [31]. These PREs occur in the upstream or intergenic region while PRE-PIK3C2B occurs in the intronic sequence. In *Drosophila* intronic PREs are predicted and validated in caudal, aPKC (Atypical Protein Kinase) and EYA [48]. The long range interaction between PRE/TRE and the cellular memory modules (CMM) is also known in *Drosophila* [49]. Though histone at the site of interaction of PRC2 is first methylated, it is observed that the inactivating histone modifications spreads along the chromatin [7], [50]. Long range effect of Fab-7 is known in *Drosophila* and position effect of silencers in yeast is known to be orientation independent affecting different promoters [12], [49], [51], [52]. Therefore the position of PRE could vary relative to its target gene(s). As we show here, PRE-PIK3C2B negatively regulates reporter gene expression when it is cloned either upstream to CMV promoter or downstream to the poly(A) signal on the vector, showing its position independent effect.

PRE-PIK3C2B is associated with PRC2 complex in HEK293T cells. Following this interaction, EZH2 subunit of PRC2 complex that has intrinsic histone methyltransferase (HMTase) activity confers H3K27me3 mark [4], [39], [53], [54]. We detect H3K27me3 modification in PRE-PIK3C2B which is lost on knock-down of *YY1* or *SUZ12*. PRE-PIK3C2B is marked by the H3K4me3, an active mark on histones which increases on knock-down of PRC2 members. Therefore PRE-PIK3C2B region carries bivalent marking, having both H3K27me3 and H3K4me3, suggesting that the gene is poised to switch between repressed and activated state [55]. The active mark on histone H3K4 is brought about by both TRX and ASH1 proteins which are TrxG proteins [53]. The PRE/TRE sequences in *Drosophila* are known to be the interaction sites for both Polycomb and trithorax complexes and have the potential to switch between repressed and activated state [1], [56], [57]. In mammalian cells, the interaction of PcG proteins leading to repression is considered as the default state which helps in the continuous proliferation in stem cells and cancers [58]. The role of TrxG proteins is implied in the reversal of this processes, for instance during differentiation of stem cells. In absence of unequivocal demonstration of the switching, Ringrose and Paro [57] suggest that the bivalent marking could be an indicator of such a transition in mammalian cells. Our observation of bivalent marking at PRE-PIK3C2B suggests the potential dual role for this region. We also compared the status of histone modification of region PRE-PIK3C2B in different cell lines in the data on whole genome histone methylation profiling [59]. The presence of both H3K27me3 and H3K4me3 modifications in the region is observed in NT2-D1 and K562 cells (Fig. S8).

The direct interaction of cloned YY1 protein and the proteins in the nuclear extract from HEK cells also shows that the repressive effect on reporter gene is mediated by YY1. However, in the gel retardation assays, the super-shift with anti-YY1 antibody was observed only when we used purified YY1, but not the nuclear extract. It could be because of the *in vivo* interactions of YY1 with other proteins leading to inaccessibility for antibody interaction.

Our results indicate that *PIK3C2B* could be the target of repression by PRE-PIK3C2B, however, long range interactions can operate on additional targets, which are being investigated. The reversal of repression is significant when both YY1 and SUZ12 are knocked down, but not when YY1 alone is down regulated. This is similar to the enhancement for example of polycomb phenotype commonly seen in only double heterozygotes of two interacting partners of the PRC complex in *Drosophila* [60], [61]. Further the eviction of the PRC complex at PRE is necessary but not sufficient to activate *PIK3C2B* expression and the recruitment of activators is also necessary. PIK3C2B being a signaling protein responds to several signals such as epidermal growth factor, platelet derived growth factors [62] and T-cell receptor signaling [63] and there are a number of transcription factors such as STAT3, p53, NF-kB and FOXO3a that regulate PIK3 expression [64].

We have demonstrated that PRE-PIK3C2B functions as a PRE in *Drosophila* and shows the characteristic pairing dependent silencing/pairing sensitive silencing in flies homozygous for PRE-PIK3C2B in PI-17 line. Even in *Drosophila*, PSS is not exhibited by all PRE/TREs but only certain PREs are known for PSS [7]. The effect of PcG members like Psc, Pcl, Scm on pairing sensitive silencing has been shown earlier and it is also observed that the extent of effect of transacting factors may not be the same for every line [65]. In concurrence with this, Psc and Scm trans-heterozygotes show reversal of pairing sensitive silencing in PRE-PIK3C2B transgenics. We detect the effect Pho mutation by genetic interaction and also at the transcription level. The absence of any significant effect of Pho in PRE-PIK3C2B flip out lines shows the specificity of the effect. The rescue of miniwhite expression that we observe in mutants of PRC 1 and 2 members is more conspicuous in the background of mutation in two members of same PRC complex like Psc and Scm, esc and E(z) or esc and su(z)12, there are cases where mutation in Psc a member of PRC1 and esc, a member of PRC2 complex lead to increase in eye pigment. This is consistent with the fact that PRC2 is involved in the recruitment of PRC1.

Though we tested interactions with many genes of the PRC complex, interaction is detected only in certain cases. Similar absence of effect of pho mutations on mini-white repression in iab-7 transgenics was reported earlier [20]. PRE-PIK3C2B with 25 repeats of PHO binding site could act as a high affinity binding site for the PcG proteins which might not abolish the interaction even in the background of decreased concentration of the trans-acting factors. The data from HEK cells lends support to this speculation as constructs carrying the 1.00 kb fragment including 25 repeats of YY1 binding site (pPRE-PIK3C2B 1kbUP/DN), shows greater repression than the one containing 11 repeats (pc(25 mer)11UP/DN). In *Drosophila* PREs are typically a few hundred base pairs and deletions reducing their length results in reduction in their repressive function [66]. Similar effect has been shown in the case of D4Z4 repeat in FSHD recently [31]. The role of repeat mediated variegation is ruled out as we do not find any effect on eye colour in PRE-PIK3C2B transgenic flies in the background of alleles *Su(var)2-5^1^*, *Su(var)3-9^6^ Su(var)2-10^1^* (data not shown). We rule out the repressive effect due to telomere position effect (TPE) as the transgene is integrated in the 5′UTR of the gene *Limpet*, about 6.7 Mb from the telomere and not in the telomere associated satellite-like sequences(TAS). DNaseI hypersensitive sites map in this region indicating that it is euchromatic in nature.

PRE-PIK3C2B shows interaction with TrxG genes zeste and brm, identified as transregulators of homeotic genes in *Drosophila* [67]. Though this may be an indirect effect, in the light of the bivalent histone mark in the region in HEK cells seen here, it suggests functional relevance. The vertebrate PRE-kr, found to interact with polycomb and trithorax group proteins showed reversal of repression in case of polycomb mutations, while only trl mutation showed a phenotype but not the other TrxG genes [29]. Recent studies have blurred the distinction between PcG and TrxG genes, as some members of each class appear to have dual function and PRE/TREs are believed to switch genes from repressive to activated state [57], [68].

In summary, we have identified a polycomb complex interacting site, PRE-PIK3C2B in the human genome through a directed search and demonstrate its function in transcription repression mediated by PRC complex. To the best of our knowledge the pairing sensitive silencing in transgenic flies is unique to PRE-PIK3C2B among the mammalian PREs reported so far. The bivalent marking on PRE-PIK3C2B and the results of the genetic interaction studies in transgenic flies, strongly suggests that PRE-PIK3C2B we describe here could indeed be a potential PRE/TRE in the human genome.

## Supporting Information

Figure S1 Complete sequence of PRE-PIK3C2B with 25 repeats of 25 mer including the YY1 binding motif (AGTGAA**GCCAT**CATGTGAGAATACC). Primers location is highlighted in yellow colour. YY1 Binding site is highlighted in red.
(EPS)

Figure S2 Interaction of proteins from HEK nuclear extract: Probe-end labeled (Oligo25 mer), Protein- HEK293 Nuclear extract, Cold (specific)- unlabeled (Oligo25 mer) was used at 100 (a),250(b) and 500 (c) fold excess. Cold (Non- specific)- Random sequence 25 mer Oligo used at 500(d) and 750 (e) fold excess.
(TIF)

Figure S3 QPCR for negative and positive controls used GADP as positive and GAPDH as negative control for ChIP with anti-YY1 and anti-SUZ12. The enrichment of PRE-PIK3C2B was significantly higher compared to the negative control in both the cases (p<0.0001 and 0.0002 for YY1 and SUZ12 respectively).
(EPS)

Figure S4 QPCR for WNT as positive and RNAP as negative control for ChIP with anti-EZH2 and anti-EED. The enrichment of PRE-PIK3C2B was significantly higher compared to the negative control in both the cases (p<0.0001 and 0.00001 for EZH2 and EED respectively).
(EPS)

Figure S5 Effect siRNA on YY1 expression in HEK293. A- qPCR for transcript level. Significant knock down (∼90%) is observed. B- Under similar conditions, protein level was observed.
(TIF)

Figure S6 Line diagram showing features of the reporter gene construct pCaSpeR. The construct contains the mini-white reporter gene under its promoter. PRE-PIK3C2B(shown as shaded arrow) was inserted upstream of the reporter gene.5′P and 3′P refer to P element present 5′and 3′to the reporter gene. The small clear arrows denote the loxP sites.
(TIF)

Figure S7 Interaction of PRE-PIK3C2B with PcG/TrxG proteins. ChIP with antibodies indicated. Input is 20% (A), 10% (B) of sonicated chromatin.
(TIF)

Figure S8 A- Image from UCSC browser, showing histone mark in the PRE-PIK3C2B region, in NT2-D1 (Pluripotent human testicular embryonal carcinoma cell line). H3K27me3 and H3K4me3 are detected in this region, showing bivalent marking (arrows). ** p<0.005. B- The histone modification in the region of PRE-PIK3C2B (202706547-202707642) in K562 cell line. Data taken from MPromDb [59]. The bivalent mark is detected in these cells also.
(TIF)

Table S1 List of top ten genes having highest density of YY1 binding site. Total refers to the total number of YY1 motifs, in the genic region, along with 15 kb upstream and downstream sequences.
(DOC)

Table S2 Details of PRE-PIK3C2B transgenic lines
(DOC)

Table S3 List of Polycomb and trithorax group mutations used in the study. ^*^Represents mutant backgrounds that showed interaction PRE-PIK3C2B transgenics.
(DOC)

Table S4 Polycomb and trithorax group of trans–heterozygote mutations used in the study. ^*^Represents mutant backgrounds that showed interaction CE-PIK2B transgenics.
(DOC)
