# Supplementary figures and images for: Identification and Validation of a Putative Polycomb Responsive Element in the Human Genome
Source: PLoS One. 2013 Jun 21;8(6):e67217. doi: 10.1371/journal.pone.0067217 (PMC3689693; doi:10.1371/journal.pone.0067217)

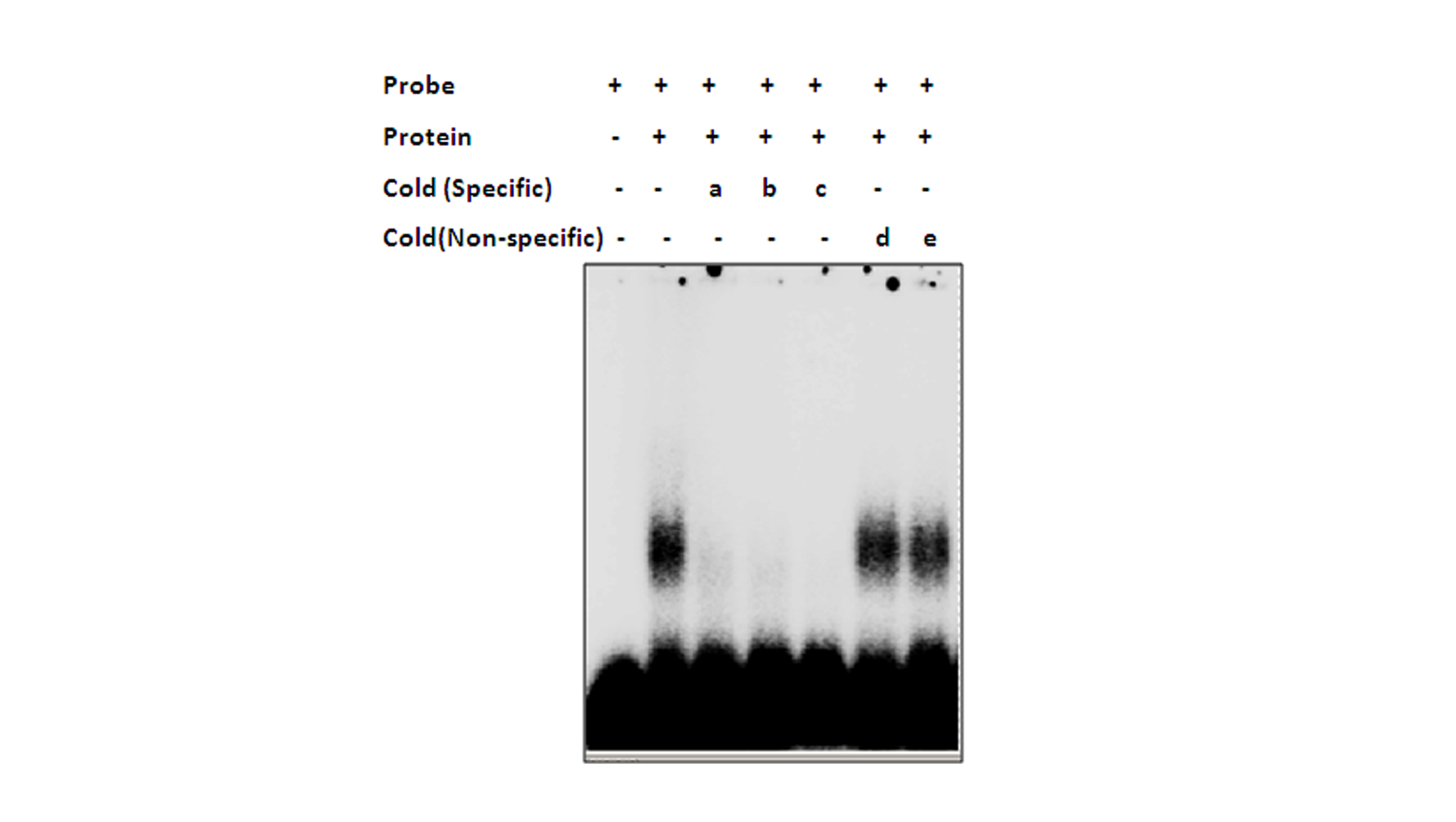

Supplement: Figure S2 — Interaction of proteins from HEK nuclear extract: Probe-end labeled (Oligo25 mer), Protein- HEK293 Nuclear extract, Cold (specific)- unlabeled (Oligo25 mer) was used at 100 (a),250(b) and 500 (c) fold excess. Cold (Non- specific)- Random sequence 25 mer Oligo used at 500(d) and 750 (e) fold excess. (TIF) [file pone.0067217.s002.tif]

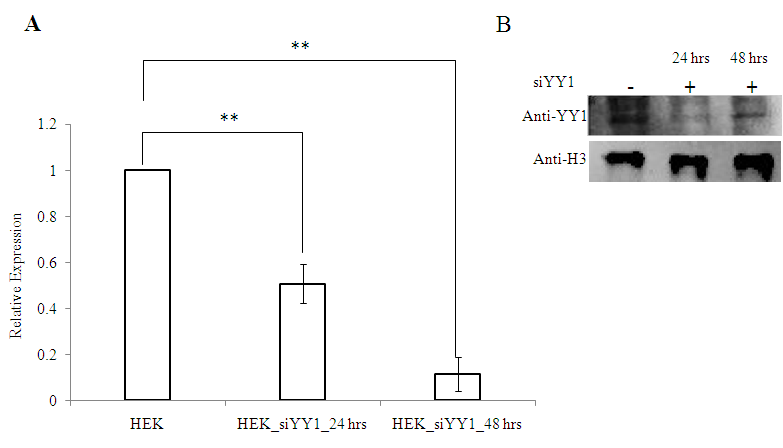

Supplement: Figure S5 — Effect siRNA on YY1 expression in HEK293. A- qPCR for transcript level. Significant knock down (∼90%) is observed. B- Under similar conditions, protein level was observed. (TIF) [file pone.0067217.s005.tif]

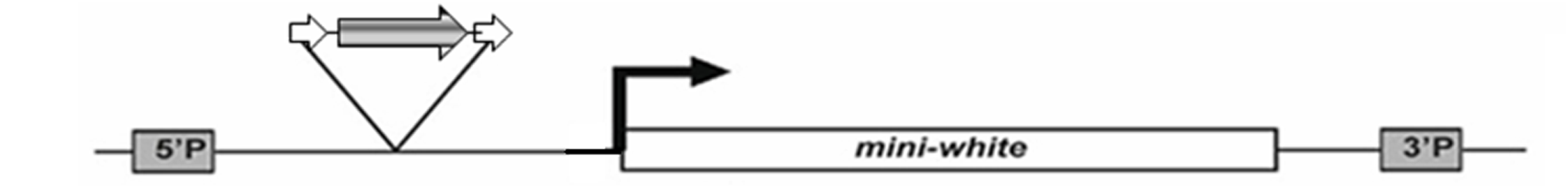

Supplement: Figure S6 — Line diagram showing features of the reporter gene construct pCaSpeR. The construct contains the mini-white reporter gene under its promoter. PRE-PIK3C2B(shown as shaded arrow) was inserted upstream of the reporter gene.5′P and 3′P refer to P element present 5′and 3′to the reporter gene. The small clear arrows denote the loxP sites. (TIF) [file pone.0067217.s006.tif]

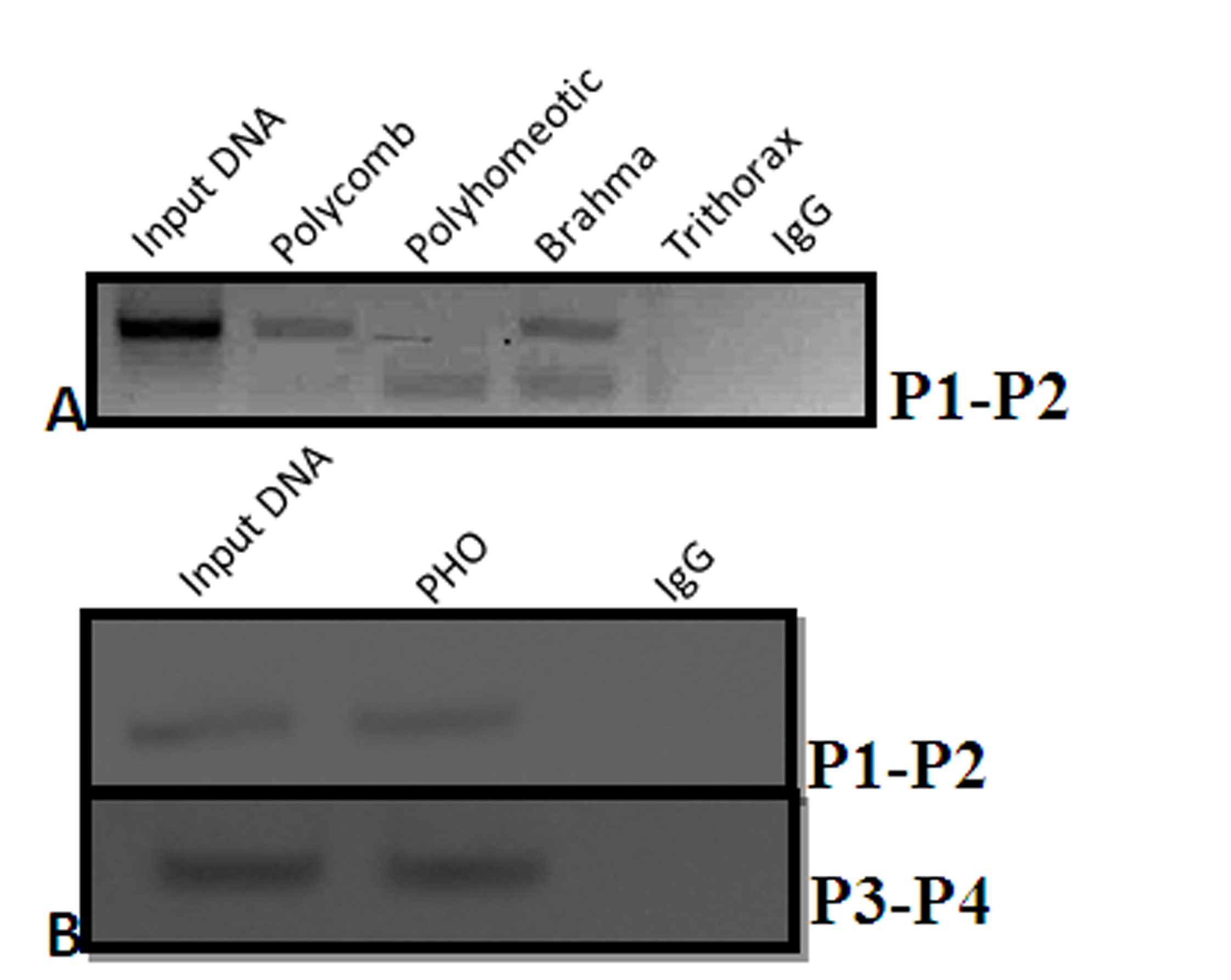

Supplement: Figure S7 — Interaction of PRE-PIK3C2B with PcG/TrxG proteins. ChIP with antibodies indicated. Input is 20% (A), 10% (B) of sonicated chromatin. (TIF) [file pone.0067217.s007.tif]

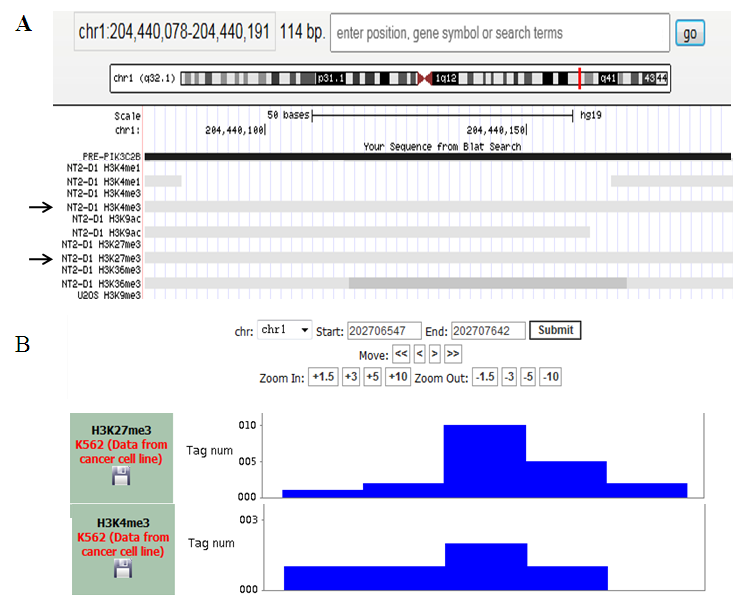

Supplement: Figure S8 — A- Image from UCSC browser, showing histone mark in the PRE-PIK3C2B region, in NT2-D1 (Pluripotent human testicular embryonal carcinoma cell line). H3K27me3 and H3K4me3 are detected in this region, showing bivalent marking (arrows). ** p<0.005. B- The histone modification in the region of PRE-PIK3C2B (202706547-202707642) in K562 cell line. Data taken from MPromDb [59]. The bivalent mark is detected in these cells also. (TIF) [file pone.0067217.s008.tif]
