# Supplementary material for: Identification and Validation of a Putative Polycomb Responsive Element in the Human Genome
Source: PLoS One. 2013 Jun 21;8(6):e67217. doi: 10.1371/journal.pone.0067217 (PMC3689693; doi:10.1371/journal.pone.0067217)
Supplement: Table S1 — List of top ten genes having highest density of YY1 binding site. Total refers to the total number of YY1 motifs, in the genic region, along with 15 kb upstream and downstream sequences. (DOC) [file pone.0067217.s009.doc]

| Gene | YY1 binding motif | |
| --- | --- | --- |
|  | Total | Frequency/10kb |
| PIK3C2B | 73 | 7.5 |
| SAMHD1 | 58 | 6.5 |
| PRKCB1 | 267 | 6.44 |
| SPTAN1 | 71 | 6.40 |
| GRB2 | 72 | 6.12 |
| RUNX1 | 178 | 6.10 |
| Bis(5 | 494 | 6.06 |
| ATP2B3 | 56 | 6.03 |
| IGFBP7 | 66 | 6.02 |
| AKAP12 | 88 | 6.01 |

**Table S1.** List of top ten genes having highest density of YY1 binding site. Total refers to the total number of YY1 motifs, in the genic region, along with 15kb upstream and down stream sequences.
