# Supplementary material for: Identification and Validation of a Putative Polycomb Responsive Element in the Human Genome
Source: PLoS One. 2013 Jun 21;8(6):e67217. doi: 10.1371/journal.pone.0067217 (PMC3689693; doi:10.1371/journal.pone.0067217)
Supplement: Table S2 — Details of PRE-PIK3C2B transgenic lines (DOC) [file pone.0067217.s010.doc]

| Stock | Chromosome | Genotype | Variegation/PSS |
| --- | --- | --- | --- |
| PI.1 | 3^rd^ | P/TM2 | **YES** |
| PI.2 | 3^rd^ | P/TM6 | **YES** |
| PI.3 | 3^rd^ | P/TM2 | NO |
| PI.5 | 3^rd^ | P/TM2 | **YES** |
| PI.7 | 3^rd^ | P/TM2 | NO |
| PI.8 | 3^rd^ | P/TM2 | **YES** |
| PI.10 | 1^st^ | P/FM7a | NO |
| PI.11 | 3^rd^ | P/TM6 | **YES** |
| P.12 | 3^rd^ | P/TM2 | **YES** |
| P.13 | 2^nd^ | P/Cyo | NO |
| PI.14 | 3^rd^ | P/TM2 | **YES** |
| PI.15 | 3^rd^ | P/TM2 | **YES** |
| PI.17 | 3^rd^ | P/TM2 | **Yes/PSS** |
| PI.18 | 2^nd^ | P/Cyo | NO |
| PI.19 | 3^rd^ | P/TM2 | **YES** |
| P.21 | 4^th^ | P/Ci | No |

**Table S2.** Details of PRE-PIK3C2B transgenic lines
