# Supplementary material for: Identification and Validation of a Putative Polycomb Responsive Element in the Human Genome
Source: PLoS One. 2013 Jun 21;8(6):e67217. doi: 10.1371/journal.pone.0067217 (PMC3689693; doi:10.1371/journal.pone.0067217)
Supplement: Table S3 — List of Polycomb and trithorax group mutations used in the study. *Represents mutant backgrounds that showed interaction PRE-PIK3C2B transgenics. (DOC) [file pone.0067217.s011.doc]

| PcG | TrxG |
| --- | --- |
| Pc^1^ | brm^2 *^ |
| Pc^2^ | mor^1^ |
| Psc^1^ | ash^1^ |
| Pcl^T1^ | ash^2^ |
| Pho^1^ | Trl^85^ |
| esc^2^ | trx^E2^ |
| Su(z)2^1^ | osa^2^ |
| ph-d^401^ph-p^602^ | z^v77h *^ |
| Pho^b^ |  |
| Su(z)12^3^ |  |
| Asx^XF23^ |  |
| Scm^R5-13B^ |  |
| E(z)^731^ |  |
| Pcl^T1^ |  |

**Table S3.** List of Polycomb and trithorax group mutations used in the study. ^*^Represents mutant backgrounds that showed interaction PRE-PIK3C2B transgenics.
