# Supplementary material for: Identification and Validation of a Putative Polycomb Responsive Element in the Human Genome
Source: PLoS One. 2013 Jun 21;8(6):e67217. doi: 10.1371/journal.pone.0067217 (PMC3689693; doi:10.1371/journal.pone.0067217)
Supplement: Table S4 — Polycomb and trithorax group of trans–heterozygote mutations used in the study. *Represents mutant backgrounds that showed interaction CE-PIK2B transgenics. (DOC) [file pone.0067217.s012.doc]

| PcG | TrxG |
| --- | --- |
| Pc^1^ & Pho^1^ | *z^v77h *^ & brm^2^ |
| Pc^2^  & Pho^1^ | z^v77h^  & mor^1^ |
| Pc^1^ &Psc^1^ | z^v77h^ & ash^1^ |
| Pc^2^ &Psc^1^ | z^v77h^ & ash^2^ |
| Pc^1^ & esc^2^ | z^v77h^ & Trl^85^ |
| Pc^2^ & esc^2^ | z^v77h^ & trx^E2^ |
| Pc^1^ & Su(z)2^1^ |  |
| Pc^2^ & Su(z)2^1^ |  |
| esc^2^& Psc^1^ |  |
| esc^2^& Su(z)2^1^ |  |
| Pc^1^ & Pcl^T1^ |  |
| Pc^2^  & Pcl^T1^ |  |
| Psc^1^& Su(z)2^1^ |  |
| Pc^1^ & Pho^b^ |  |
| Pc^1^ & ph-d^401^ph-p^602^ |  |
| *Psc^1^ & Scm^R5-13B^ |  |
| *E(z)^731^ & esc^2^ |  |
| *esc^2^ & Su(z)12 |  |
| *esc^2^ & Psc^1^ |  |
| *esc^2^ & Pcl^T1^ |  |

**Table S4**. Polycomb and trithorax group of trans–heterozygote mutations used in the study. ^*^Represents mutant backgrounds that showed interaction CE-PIK2B transgenics.
